# Culinary medicine and community partnership: hands-on culinary skills training to empower medical students to provide patient-centered nutrition education

**DOI:** 10.1080/10872981.2019.1630238

**Published:** 2019-06-27

**Authors:** Brandon Pang, Zoe Memel, Carmel Diamant, Emily Clarke, Sherene Chou, Harlan Gregory

**Affiliations:** a Department of Medical Education, Keck School of Medicine of USC, Los Angeles, CA, USA; b Department of Culinary Training, LA Kitchen, Los Angeles, CA, USA; c Department of Medical Education, Keck School of Medicine of USC, Los Angeles, CA, USA; d Department of Pediatrics, Keck School of Medicine of USC, Los Angeles, CA, USA

**Keywords:** Culinary medicine, students, nutrition, medical education

## Abstract

Given the economic burden and numerous morbidities associated with obesity and poor dietary choices, it is increasingly important for medical students to receive education on nutrition and preventive medicine so that they are equipped to advise patients about healthy lifestyle choices. Currently, 71% of US medical schools do not reach the minimum benchmark of 25 hours of nutrition education set by the National Academy of Sciences. In order to improve the quality and quantity of nutrition education at the Keck School of Medicine of USC (KSOM), medical students and faculty have partnered with LA Kitchen (LAK), a local teaching kitchen, and the Wellness Center at LA County Medical Center (LAC+USC). They developed a hands-on preclinical culinary and nutrition course that aims to teach students practical skills and knowledge that they will be able to apply to their own lives and pass onto patients. Following the completion of the first three years of the course (2016–2018), analysis suggests that the class was well-received and has improved students’ nutrition knowledge, confidence in lifestyle counseling, and personal culinary skills. Given these highly encouraging observations, the project is currently aimed at incorporating nutrition education more broadly into the required preclinical curriculum at KSOM.

## Introduction

In a medical system increasingly burdened by obesity and its comorbidities, the onus is on the medical profession to educate the general population on proper nutrition and other preventive health practices. In 1985, a report by the National Research Council’s Committee on Nutrition in Medical Education recommended 25 hours of required nutrition instruction by medical schools [1]. Given the increasing scope of medically relevant nutrition knowledge and an increasingly overweight patient population in the intervening 33 years, it is reasonable to assume that this recommendation should be taken as a minimum requirement for medical schools. However, a recent study of 121 medical schools conducted by the University of North Carolina indicates that only 29% of medical schools provide the minimum recommended 25 hours [2,3]. Additionally, only 18% of schools reported that their curriculum included a dedicated nutrition course. The consequences of this are far-reaching, as the literature supports that many doctors do not feel comfortable dealing with overweight and obese patients, nor are they comfortable with their clinical nutrition skills overall [4,5]. With obesity and lifestyle-related disease on the rise, it is a matter of critical importance to address the shortcomings in nutrition education for future physicians.

The Keck School of Medicine, like the majority of medical schools in the USA, currently does not meet the recommended 25 hours of nutrition education. In 2016, a group of medical students at KSOM created a Culinary Medicine course in order to introduce more clinical nutrition training into the curriculum. Although numerous medical schools have similarly created independent culinary medicine courses [6], KSOM’s approach has been a uniquely student-driven and student-centered initiative. Overall, the Culinary Medicine course had three main goals that guided the framework of the class:

1. Provide Keck students with a hands-on approach to nutrition education through cooking-based modules centered around teamwork.
2. Create a new way for students to connect with the LA County community and better understand the health disparities facing this population through the lens of food and cooking.
3. Equip students with the skills and experience needed to counsel and support their patients in making healthy and positive dietary changes to best treat their chronic diseases.

## Methods

### Conception of the course

We developed this course in partnership with LA Kitchen, a local nonprofit teaching kitchen that oversees multiple social welfare programs dedicated to enhancing the community through culinary job training of marginalized populations and reduction of food waste. A critical aspect of course development was our collaboration with LAK’s dietitian and chefs to create a more cohesive culinary nutrition curriculum. In partnership, we developed an interdisciplinary class curriculum combining aspects of nutrition, community engagement, and culinary education. We instructed students in the basic aspects of nutrition while also allowing students to engage with the local patient population through a community partner. Adding a service-learning component served as a valuable way to connect our students to the community [6,7]. Furthermore, Ring et al. state that ‘service learning has been linked to students developing attributes of altruism, more favorable academic outcomes, [and] increased interpersonal and community skills … which are [all] critical for physicians to address complex public health problems such as obesity’ [7]. With this in mind, we aimed to incorporate community engagement as a core value of the course. We did this initially by partnering with LAK and then later collaborated with the Wellness Center which directly serves the patients of LAC+USC Medical Center. The Wellness Center is a non-profit organization that aims to provide culturally sensitive preventive services and resources to address patients’ root causes of disease and improve health outcomes in underserved areas. Some of their services include free exercise classes, cooking courses, legal assistance and housing resources for residents of Los Angeles County [8].

A 6-week pilot program taught at LAK was launched in Fall 2016 as a selective course available to 15 second-year medical students. Although there were more barriers to entry compared to other selective courses (including an application essay), interest was notably very high, with many more applicants than positions available. Applications were judged based on the perceived level of interest in the course, and considerable effort was made to select a diverse group of students with a varied background in nutrition and cooking. Students’ experience with nutrition and cooking ranged from no experience cooking and having never taken a nutrition course to students who had a bachelor’s degree in nutrition and extensive experience cooking for themselves or family. Based on the application essays, students’ motivations for taking the course included a desire to: improve their personal or their family members’ health, improve their ability to counsel patients in their continuity clinics, and overcome their feelings of hypocrisy when telling patients to ‘eat healthy’ when they did not know how to do so themselves. Students also wanted to explore the economic realities of healthy diets, to dispel the myth that healthy food is more expensive than fast food, and to better understand the utility of vegan and plant-based diets in disease treatment and prevention.

### Culinary medicine curriculum

**Figure 1.**
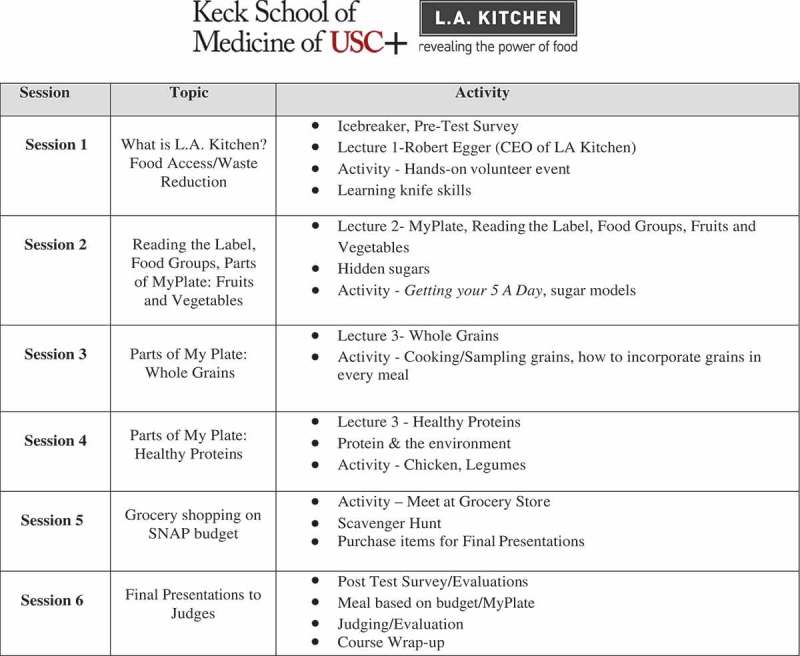
Culinary medicine course syllabus for fall 2016–2017. Mondays, 3:00–5:30 PMLocation: L.A. Kitchen, 230 W. Ave 26 Los Angeles, 90031Instructors: Dr. Gregory Harlan, Sherene Chou, MS, RD, Chef Theresa Farthing

**Figure 2.**
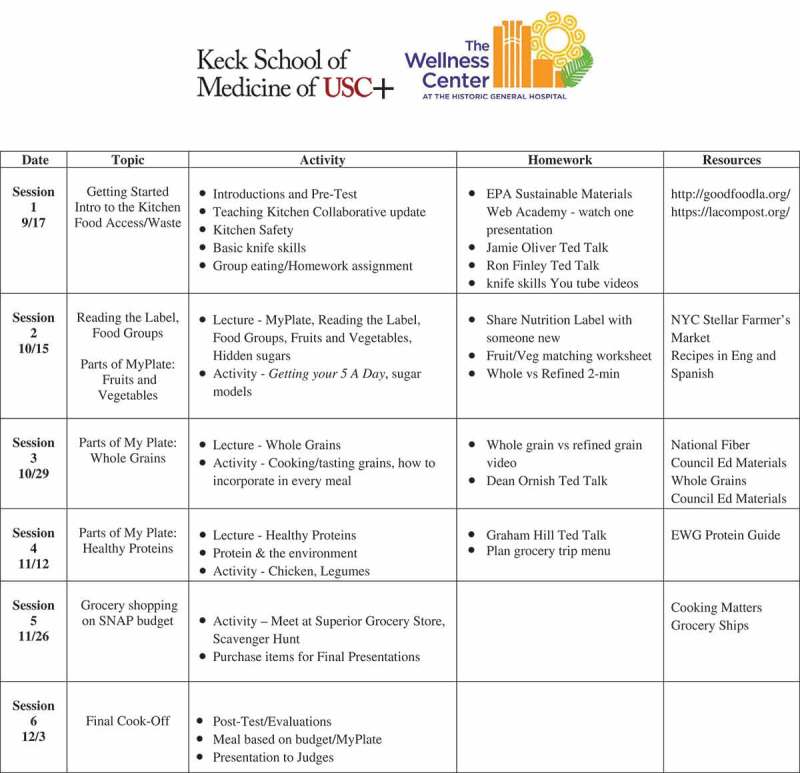
Culinary medicine course syllabus for fall 2017–2018. Mondays, 1:15–3:45 PMLocation: Teaching Kitchen at LAC+USC Wellness CenterInstructors: Gregory Harlan, MD MPH & Sherene Chou, MS RD

In order for students to learn more about nutrition through multiple lenses, each session was jointly taught by a physician, a registered dietitian, and a chef. Additionally, medical students contributed to the development of the curriculum, as we attempted to be as student-centered as possible in its creation. The 2016 pilot focused on the management of the top three diseases in Los Angeles County: hypertension, diabetes, and heart disease. In 2017 and 2018, the course shifted focus to be based on the MyPlate guidelines. The lesson topics were divided into six weeks, as outlined in Figures 1 and 2. Each week students were instructed in case-based disease pathophysiology, appropriate dietary management and meal preparation on a limited budget. Evidence-based guidelines and scientific literature in the form of journal articles, patient cases, documentaries and interactive websites were used to supplement each week's lesson plan. Instruction in basic kitchen skills was also provided which included basic knife technique, food safety and sanitation, and the fundamentals of cooking. Additionally, in deciding on weekly recipes, the USA Department of Agricultures (USDA) Supplemental Nutrition Assistance Program (SNAP) ‘Just Say Yes to Fruits and Veggies’ [9] recipe guide was frequently used.

Throughout the course, we aimed to teach students how to translate their new culinary skills into tools to counsel patients and improve their own confidence in cooking healthy meals for themselves. The typical lesson plan involved three sections. The course physician would lead a discussion on how the disease, and later MyPlate topic, related to patients’ health for 30 min, with students contributing personal patient anecdotes. Next, the registered dietitian and chef provided strategies to tailor a patient’s diet for that specific disease as well as motivational interviewing techniques that could be utilized to counsel a patient on healthy eating. The group would then learn specific cooking techniques throughout a 90-min cooking lesson followed by 30 min of communal eating and a session debrief. Students were often divided into groups of four and were each assigned a component of the meal to make, receiving feedback while they cooked. Examples of lessons included: how to replace salt with flavorful spices, making a basic roux, cutting vegetables safely, and improving basic knife skills.

We hoped to make the class as practical and hands-on as possible so that students could learn concrete skills that they could provide to their patients. Every lesson plan ended with students sharing the dishes they made with the class. For the class final, students were assigned to teams and applied lessons learned throughout the course to create a meal plan according to specific scenarios. As a way of highlighting food access issues, each team was expected to design a healthy meal plan based on MyPlate guidelines while facing income restraints similar to those faced by Supplemental Nutrition Assistance Program (SNAP/CalFresh) recipients [10]. For example, the 2018 cohort was tasked with creating a menu for a family of four which would include three meals and two snacks for only $22. Students were challenged to not only create a low-cost, healthy meal plan, but to cook the dinner meal and explain the health benefits to a panel of student and faculty judges.

### Modifications and improvements to the course over time

Since the fall of 2016, three cohorts have successfully completed the culinary medicine class. The first two groups of students were taught in partnership with LA Kitchen. In the fall of 2018, we moved the course to the Wellness Center teaching kitchen at LAC+USC Medical Center. This kitchen is across the street from the medical school and more convenient for students and physicians to access. It also upholds our mission of working with community partners in order to allow students the ability to engage with members of the surrounding neighborhood. Based on feedback at the end of each group, the lesson plans were modified to a small degree to improve the course. Figures 1 and 2 illustrate the improvements made in the course from 2016 to 2018 with respective curriculum breakdowns. We made considerable efforts to enhance the course based on student feedback, with some of the highlights, as seen in Figure 2, including changing the course from disease-based topics to incorporating more focus on MyPlate guidelines (the current USDA nutrition guide) as well as adding a focused tour of a local grocery store and more pre-class assignments. Additionally, the pre and post survey was slightly modified after the 2016 cohort with different confidence questions and a shorter version of the knowledge questions to reduce exam fatigue and increase relevance (Appendix 2).

### Course evaluation and measurement of student improvement

On the first day of each cohort's class, a pre-test survey was given to each group. The same pre and post-test survey was given to each year’s cohort. As mentioned, the 2016 survey was slightly modified for the 2017 and 2018 cohorts (included in Appendices 1 and 2). The surveys were created by the course’s registered dietitian with questions assessing students’ knowledge in nutrition, their ability to identify various foods by visual inspection, and their confidence in cooking and nutrition counseling. Additionally, all students provided course feedback with ways to improve the course and rate their overall satisfaction with the class through an anonymous survey at course completion.

## Results

### Course evaluation

There was a 100% completion rate for both the pre and post-test surveys for all three cohorts. Fifteen students participated in the 2016 class, 16 students in 2017 and 16 students in the 2018 class. The three primary objectives measured were students’ subjective confidence in nutrition counseling, nutrition knowledge in culinary technique and nutrition, and food identification skills. We conducted Wilcoxon Signed Rank tests for each class individually as well as combined to assess the effect of the course by class. As seen in Table 1, students demonstrated a statistically significant improvement in their nutrition knowledge level with moderate to large effect sizes (2016: r = −.49; 2017: r = −.55; 2018: r = −.49). Students' ability to identify food by visual inspection only showed significant improvement with moderate to large effect size in class years 2017 (r = −.56) and 2018 (r = −.40). The largest impact of this course was boosting students’ confidence in counseling patients and cooking healthy meals across all three years where large effect sizes were achieved from pre-course survey to post-course survey (2016: r = −.62; 2017: r = −.63; 2018: r = −.60). On course evaluation, students gave the first course an overall satisfaction rating of 4.87/5, the second course a 5/5 and the third course a 5/5.

**Table 1.** Pre-course and post-course survey measuring students’ confidence, nutrition knowledge, and food identification skills.

	Year	2016^a^	2017^b^	2018^b^
	Students (n)	15	16	16
Confidence	Pre-Course Median	2.5	2.19	2.38
	Post-Course Median	3.33	2.88	2.69
	z value	−3.42	−3.54	−3.42
	**p value^d^**	**0.001**	**<.001**	**0.001**
	r	−0.62	−0.63	−0.60
Nutrition Knowledge Score^c^	Pre-Course Median	57.69	62.5	50
	Post-Course Median	69.23	78.13	62.50
	z value	−2.741	−3.09	−2.744
	**p value**	**0.006**	**0.002**	**0.006**
	r	−0.49	−0.55	−0.49
Food Identification^c^	Pre-Course Median	75	55	68.75
	Post-Course Median	70.00	72.5	75.00
	z value	−0.035	−3.186	−2.202
	**p value**	**0.972**	**0.001**	**0.028**
	r	−0.006	−0.56	−0.40

^a^Student confidence recorded on 0–4 Scale; survey available in appendix 1 ^b^Student confidence recorded on 0–3 Scale; survey available in appendix II ^c^Both Nutrition Knowledge and Food Identification on a scale from 0–100 ^d^P Value <0.005 considered statistically significant

### Adjustments to KSOM preclinical nutrition education

Prior to the development of the USC Culinary Medicine selective, preclinical nutrition education totaled 4 hours across gastroenterology, cardiology, and endocrinology system blocks. Moreover, lectures in which nutrition were taught were usually not specifically dedicated to the topic of nutrition itself. Instead, nutrition was mentioned in the management of the disease-specific entity being discussed. Students participating in this selective received an additional 15 hours of nutrition education, and our current goal is to integrate nutrition and dietary education more broadly into the required preclinical curriculum. At the time of this writing, the quantity of required preclinical nutrition education has doubled to 8 hours over two years. Members of this team helped introduce new lectures into the first-year curriculum including two lectures during the renal block on sodium reduction and potassium-rich foods, a lecture on carbohydrates and sugar reduction during the cardiology block, and a lecture on various types of evidence-based diets providers can discuss with patients.

## Discussion

### Outcomes and quality assurance

An important consideration is not merely the quantity, but the quality of instruction. Student evaluations suggest that the course has been well-received and effective in improving student knowledge and general self-confidence regarding nutrition (Table 1). Studies are currently ongoing which track student attitudes and knowledge over time as nutrition becomes more heavily integrated into the preclinical and clinical curriculum.

In response to student feedback, we integrated more pre-reading and hands-on cooking homework to provide students with more time to understand concepts and apply new culinary techniques to their everyday lives. This was especially well-received by those with little to no prior nutrition knowledge. Some of the resources added include chapters of healthy cookbooks, scientific publications on nutrition, food-based documentaries, and local newspaper articles on nutrition initiatives. Aspects of the curriculum that were thought by students to be too esoteric or unrealistic (including recipes thought to be unpalatable to the local patient population) were replaced by more practical instruction in basic kitchen skills, serving sizes, and more accessible recipes. We believe the shift to the MyPlate framework has made the course more practical and easier for students to understand nutrition concepts as illustrated by a statistically significant improvement in students’ food identification skills with moderate to large effect size in 2017 (r = −.56) and 2018 (r = −.40) after the MyPlate framework was introduced. Both students’ confidence in counseling patients across all three years (2016: r = −.62; 2017: r = −.63; 2018: r = −.60) and their knowledge scores continued to show statistical improvement with the new MyPlate intervention (2016: r = −.49; 2017: r = −.55; 2018: r = −.49). Additionally, a concerted effort was made to recreate the conditions under which the local patient population would have to prepare meals. Food access and affordability will continue to be a major focus of the curriculum. Anecdotally, students who have graduated from the course tell us that they have continued cooking for themselves while on their clinical clerkships and practiced their nutrition counseling with colleagues, patients, and family members.

Students praised the intense involvement of our dietitian and chef. This fits well with the desire to promote more interdisciplinary coursework in medical schools and supports the fact that expertise outside of traditional medical channels is needed to support medical student education and improved patient outcomes. We hope that other schools reach out to their nutrition counterparts to involve them in future course offerings.

### Limitations, resources, and goals

The influence of this nutrition intervention on the student body has been limited to 47 students so far. Although the number of preclinical nutrition lecture hours has been trending up, a disconnect remains between the experience of students who take the selective course and the student body at large. The ability to expand the selective course to include more students is currently limited by finances and logistics. Efforts are currently underway to expand the number of clinical nutrition experiences available to students, through standardized patients and various teaching sessions in development. Based on what we have learned from this pilot program, there is active discussion of implementing a hands-on nutrition curriculum for Year I medical students. By increasing the availability of the course to as many students as possible, we hope to maximize any potential benefits to the community and our patients.

An important limitation to consider when evaluating the survey data is the testing effect that may have occurred due to the same survey being administered to students before and after the course. Given the extended length of time between the initial administration of the survey approximately 3 months before the second survey, we believe that a large proportion of the questions answered correctly were due to new knowledge acquired as opposed to any practice effect as a result of the pretest. We plan to carefully consider how best to perform the post-course exam to eliminate any practice/testing effect while still providing an accurate assessment of objective and subjective improvement as a result of the course. Despite this limitation, we believe the overall positive change in confidence and knowledge throughout all three class years reflects the positive impact this course has had on students’ confidence in cooking and counseling patients on nutrition.

Another limitation of the course is that although students report a subjective improvement in the knowledge that is mirrored in examination performance, it is difficult to assess how effectively they will translate this knowledge into clinical practice, if at all. Even if students were to apply the material they learned in a clinical setting, we currently have limited methods of assessing any improvements in patient outcomes. Such assessment would likely require a large population study that is beyond the scope of this project and given the limited number of students who have currently taken the course, any effect is likely to be negligible at a population level.

### Future directions

In order for hands-on nutrition courses to become a required aspect of the medical school curriculum, there needs to be action on both a local and national front. From the nationwide perspective, reinforcing the importance of preventative medicine and nutrition education within medical schools’ teachings can be done by including a new section on the US Medical Licensing Exams (USMLE) that assesses students’ understanding of basic nutrition and motivational interviewing. Additionally, we believe that the Liaison Committee on Medical Education (LCME) and the Association of American Medical Colleges (AAMC) should consider adding a minimal amount of nutrition education to their curriculum requirements. These actions would provide both administrators and educators with the motivation and justification to allocate time and resources to the creation of programs similar to this one.

On a more local level, one of the greatest challenges to creating a required hands-on nutrition course for all students is the time required and the funds needed to launch an interactive and time intensive initiative. On reflecting on our course’s journey, our recommendations are the following:

1. Illicit interest and feedback on the course idea from faculty, administration and students campus-wide prior to starting the pilot program. We found that many faculty and students were interested in the topics of nutrition and culinary medicine and were interested in creating similar courses. There is more power in numbers when creating a new program.
2. Take student feedback seriously and adjust the curriculum accordingly. You can strengthen your program each year as students provide guidance on how it can improve.
3. Apply for local grants and elicit donors who would be interested in supporting a culinary medicine program in the long term. A difficult challenge we faced was continued funding. Identify community partners and resources within your existing faculty and clinical enterprise. This can include researchers, dietitians, educators, administrators and clinicians.

Start small when considering making a course required. Although our long-term goal is to make our six-week culinary medicine course required for all students, we aim to initially expose all students to nutrition education with a 4-hour workshop on culinary medicine and exposure to some motivational interviewing for all Year I medical students. We plan to increase the minimum hours each year.

Since concluding the third culinary medicine course, USC has become a member of the Teaching Kitchen Collaborative [11], started by the Harvard T. H. Chan School of Public Health and the Culinary Institute of America. The focus of this collaborative is to ‘foster and create “learning laboratories” across multiple organizations that implement nutrition education, culinary instruction, enhanced movement and exercise, mindfulness training, and health coaches’ [11]. In joining a coalition of universities and companies dedicated to creating initiatives that allow both health professionals and the general population to learn more about nutrition and positive lifestyle behaviors we can shift the trends in our nation away from obesity and stagnation. We have added over 5 hours of preclinical lectures on nutrition for the entire first-year class, hired a registered dietitian to lead healthy eating on a budget workshops, and are currently working on a standardized patient case for all second-year medical students to practice their motivational interviewing techniques.

We have created a student-centric clinical nutrition and culinary skills course that aims to address a pressing issue facing medical education today: the need for improved nutrition training among medical students. By working with LAK and the Wellness Center, we have engaged our community partners and satisfied students’ desires to become more integrated with our surrounding community and patients. Our hope is that this will result in more well-rounded physicians and improve patient care. In addition, we hope that our efforts create a larger trend to integrate nutrition, diet, and lifestyle education more broadly into medical education.
